# Clinical applications of remote ischaemic preconditioning in native and transplant acute kidney injury

**DOI:** 10.1007/s00467-014-2965-6

**Published:** 2014-10-04

**Authors:** Kristin Veighey, Raymond MacAllister

**Affiliations:** 1 UCL Centre for Clinical Pharmacology & Therapeutics, University College London, The Rayne Building, University Street, London, WC1E 6JJ UK; 2 UCL Centre for Nephrology, University College London, London, UK

**Keywords:** Ischaemia–reperfusion, Acute kidney injury, Transplantation, Remote ischaemic preconditioning, Therapeutic benefits

## Abstract

Ischaemia–reperfusion (IR) injury is a composite of the injury sustained during a period of reduced or absent blood flow to a tissue or organ and the additional insult sustained upon reperfusion that limits the amount of tissue that can be salvaged. IR injury plays a central role in both native and transplant acute kidney injury (AKI). Native AKI is associated with increased morbidity and mortality in hospital inpatients, and transplant AKI contributes to graft dysfunction, ultimately limiting graft longevity. In this review, we discuss the potential therapeutic benefits of a cost-effective and low-risk intervention, remote ischaemic preconditioning (RIPC), and its applicability in the prevention and reduction of AKI.

## Introduction

When an organ or tissue is rendered ischaemic, there is inevitable cell death and tissue injury, the extent of which can be limited by timely reperfusion. However, paradoxically, an additional injury occurs upon reperfusion which limits the amount of tissue that can be salvaged. This composite injury is termed ‘ischaemia–reperfusion (IR) injury’.

In both native [1–3] and kidney transplant [4] acute kidney injury (AKI), IR injury plays a significant role. The pathogenesis of AKI, regardless of aetiology, results in a degree of IR injury, the majority of which affects the vulnerable tubular epithelium. Native AKI is associated with the subsequent development of chronic kidney disease (CKD) [5, 6] and increased mortality in patients admitted to hospital [7]. In renal transplantation, despite an improvement in 1-year kidney transplant survival, long-term allograft survival has not altered significantly in recent years [8–10]. IR injury at the time of transplantation is associated with an increased risk of acute rejection, delayed graft function and poor overall graft function [11]. Therefore, strategies to reduce IR injury at the time of transplantation may be the best therapeutic intervention to increase graft longevity.

Ischaemic preconditioning is an intervention targeted against IR injury whereby brief, non-lethal periods of ischaemia activate an innate response that confers protection against a later prolonged and thus potentially lethal period of ischaemia. Ischaemic conditioning may be applied before (preconditioning), during (perconditioning) or following (postconditioning) ischaemia and may be applied remotely. In this review, we discuss the potential of remote ischaemic preconditioning (RIPC) in protecting against native and transplant AKI. The therapeutic potential of this simple, low-cost protective strategy has attracted much attention in recent years, with a number of large clinical trials about to report their findings, including one in renal transplantation.

## Ischaemia–reperfusion injury

Ischaemia–reperfusion injury was first described in 1960 by Jennings et al. who demonstrated that myocardial infarct size in the dog following 24 h of ischaemia alone was similar to that after 30 min of ischaemia followed by 60 min of reperfusion [12]. A variety of experimental interventions to specifically modify the reperfusion phase of IR have been shown to reduce IR injury, further establishing the injurious nature of reperfusion.

Strategies to limit clinical ischaemic injury have mainly focussed on timely reperfusion, including interventions such as primary coronary intervention, thrombolysis for stroke and reduction of both warm and cold ischaemic times in transplantation. However, given that a significant proportion of tissue dysfunction and cell death following IR injury is attributable to reperfusion injury and that there has arguably been maximal optimisation of therapeutic techniques and their timing (within the current framework of healthcare delivery), attention has turned towards interventions which specifically target IR injury, either to enhance resistance to ischaemia and/or to reduce reperfusion injury. One such strategy is ischaemic preconditioning.

## Ischaemic preconditioning

Ischaemic preconditioning is a whole body innate reflex that protects against subsequent IR injury and is activated by brief, non-lethal periods of tissue or organ ischaemia. The phenomenon was first described in 1986 by Murray et al. who demonstrated that a series of 45-min periods of circumflex coronary artery occlusion, separated by 5 min of reperfusion, could significantly reduce myocardial infarct size in dogs following subsequent prolonged ischaemia [13]. The magnitude of the effect size observed was much greater than that found with any pharmacological agent, and the effect was widely reproducible and subsequently demonstrated in many different animal models and species, including chicken, pig, rat, dog, mouse and sheep [14].

Following on from Murray et al.’s [13] discovery, Pryzlenk et al. demonstrated that ischaemic conditioning could be applied remotely [15]. These authors found that brief periods of ischaemia applied to one vascular bed could remotely protect another—in this case circumflex artery preconditioning protected the anterior descending coronary artery territory from injury following a subsequent prolonged occlusion [15]. Subsequent studies established that the preconditioning stimulus could be applied to a different organ, with protection spreading to remote organs, including the heart, brain and kidney. Now termed ‘remote ischaemic preconditioning’, this intervention gained potential clinical applicability with the discovery that the ischaemic preconditioning stimulus could be applied non-invasively in humans using a blood pressure cuff placed on a limb and inflated above systolic blood pressure to induce limb ischaemia [16].

The protective effects of ischaemic preconditioning have been demonstrated to occur in two ‘windows’, with the initial period of protection occurring immediately following the preconditioning stimulus and lasting for between 1 and 4 h [17–19], and the onset of a delayed or ‘second window of protection’ occurring at 24 h following preconditioning, and lasting for between 24 and 72 h [18, 20, 14]. However, it should be noted that timely reperfusion remains a requirement even despite ischaemic preconditioning [13], with the latter delaying rather than abrogating the onset of cellular death. The discovery that preconditioning could be activated both non-invasively and remotely led directly to potential clinical applications of this therapy. Initial interest in cardioprotection has extended to virtually all organ systems that may be subjected to IR injury in the clinical setting, including not only cerebrovascular disease but also many forms of surgery.

## Mechanisms of protection of RIPC

From its inception, researchers have postulated that RIPC relies on three components: local mediators which initiate (or trigger) the preconditioning cascade, humoral and/or neural factors which transfer protection systemically from the remote site and end-effectors which confer this protection to the threatened organ or tissue.

### Triggers

Certain factors, termed ‘trigger factors’, are released locally at the time of preconditioning ischaemia and include adenosine, bradykinin and endogenous opioids. These triggers initiate the cascade of protection locally by activating G-protein-coupled receptors [14] and thereby promoting the recruitment of protein kinase mediators (such as PI3K, ERK/MAPK, PKC and JAK/STAT) [21–23].

### Signal transduction

The mechanism by which the protective signal is transferred systemically from the area of index ischaemia has been the subject of some debate. Evidence for the involvement of a humoral factor is supported by the observation that protection can be transferred by the transfusion of serum from a rabbit that has undergone ischaemic preconditioning to one which has not [24, 25]. This factor is believed to be a protein that is heat stable, dialysable and of a size less than 15 kDa [26, 27]. In pigs, RIPC applied to the recipient animal conferred protection against IR injury to the denervated donor heart during transplantation, again supporting a humoral hypothesis [28]. Attempts to identify this circulating factor have proved challenging. However, recently, stromal cell-derived factor-1 (SDF-1α or CXCL12), a cardioprotective chemokine of 10 kDa that is induced by hypoxia, has been demonstrated to be upregulated following RIPC in rats. The resultant cardioprotection was blocked in rats treated with AMD3100, a highly specific inhibitor of CXCR4, the target receptor for SDF-1α [29].

Neurogenic mechanisms of signal transfer have also been suggested. In rats, Dong et al. demonstrated that femoral nerve section abolished the effects of limb IPC [30]. Local injection of adenosine into the nerve produced a similar protection to that of IPC, whereas intravenous injection of adenosine had no effect. Administration of an adenosine antagonist partially abolished the effects of IPC [30]. In a rat myocardial infarction model, hexamethonium (an autonomic antagonist) abolished the protection by RIPC achieved by mesenteric artery occlusion [31]. The autonomic ganglion blocker trimetaphan has been shown to inhibit RIPC in a human model [19].

The humoral and neuronal pathways may work in series to spread protection systemically. Lim et al. demonstrated that in mice, femoral vein occlusion or femoral and sciatic nerve resection abolished the protective effects of RIPC, implicating both humoral and neural pathways [32].

### End-effectors

Ischaemic preconditioning activates at least three main salutatory pathways, i.e. the cyclic guanosine monophosphate/cGMP-dependent protein kinase (cGMP/PKG) pathway [33], the reperfusion injury salvage kinase (RISK) pathway [34, 35] and the survivor-activating factor enhancement (SAFE) pathway [36]. There is a degree of overlap between these pathways, in particular where they converge in mitochondria [37]. In the mitochondria, although there is some uncertainty regarding the mechanism, the potassium-dependent ATP (K_ATP_) channel is activated, leading to closure of the mitochondrial permeability transition pore (mPTP). Closure of the mPTP prevents the influx of ions through this channel, thus preventing mitochondrial rupture and cell death by apoptosis.

## The second window of protection—delayed or late IPC

Ischaemic preconditioning initiates a complex genomic and proteomic response that is thought to underpin the late phase of protection. This includes regulation of anti-apoptotic and anti-inflammatory gene transcription, which is likely to be responsible for the second window of protection [38, 39]. Later phase protection requires the synthesis of inducible nitric oxide synthase, heat shock proteins or cyclo-oxygenase-2, secondary to the upregulation of genes for these factors. These then act locally via the mPTP or K_ATP_ channels to induce a state of protection [40].

## The role of the innate immune system

Activation of the innate immune system by non-lethal periods of ischaemia may be mediated by germline-encoded toll-like receptors (TLRs) and may contribute to the development of ischaemic tolerance. Activation of feedback inhibitors of inflammation following an ischaemic insult effectively renders the tissue immunosuppressed; consequently, the inflammatory response to a subsequent lethal insult is attenuated [41]. In mice, deficiency of TLR4 is associated with a reduction in myocardial IR injury in mice [42], but the absence of a functional TLR4 has been demonstrated to block IPC [43, 44].

## Pathophysiology of renal IR injury

During ischaemia, renal epithelial cells are deprived of ATP and are therefore unable to maintain essential homeostatic processes. This ultimately leads to cell death by apoptosis or necrosis [45] if timely reperfusion does not occur. Although any segment of the nephron may be affected, the cells most vulnerable are in the renal proximal tubule and distal medullary thick ascending limb of the loop of Henle [46, 47]. The factors contributing to the vulnerability of these cells to ischaemia are high metabolic rate, required for ion transport, and a limited capacity for anaerobic metabolism. Additionally, marked microvascular congestion and hypoperfusion have been found in this region which persists despite restoration of cortical blood flow, therefore contributing to prolonged ischaemic injury [47]. Proximal tubular cell injury leads to afferent arteriolar vasoconstriction by tubuloglomerular feedback, luminal obstruction and backleak of filtrate across injured cells, with resultant ineffective glomerular filtration and a profound drop in the glomerular filtration rate (GFR). Endothelial cell injury and endothelial dysfunction are primarily responsible for this phenomenon, known as the extension phase of AKI [48].

Ischaemic injury results in the loss of the apical brush border of proximal tubular cells. Disrupted microvilli detach from the apical surface, forming membrane bound blebs that are released into the tubular lumen. The detachment and loss of tubular cells, in combination with brush border vesicle remnants, cellular debris and uromodulin, result in tubular casts which may cause obstruction [3, 47]. Necrotic cell death is rare, but it may occur in the highly susceptible outer medullary regions. Conversely, apoptosis may be seen in both proximal and distal tubular cells. Apoptosis has been demonstrated in distal tubular cells during nephrotoxic AKI and also in donor kidneys before transplantation—in one study this was found to be associated with delayed graft function [49]. In addition to tubular cell injury, podocyte dysfunction may occur, with foot process effacement and loss of slit diaphragm integrity, and resultant proteinuria [50].

**Fig. 1:**
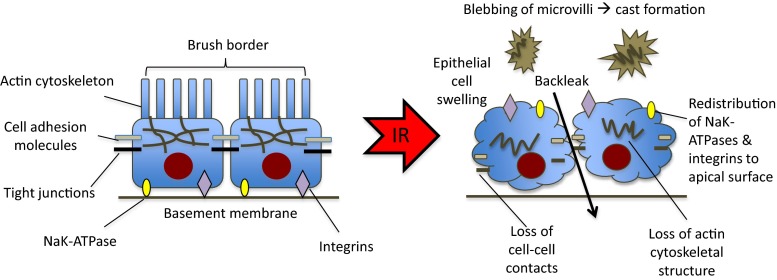
Diagram illustrating the effects of ischaemia–reperfusion (*IR*) injury on the renal tubular epithelium

Alteration in the cell cytoskeleton also contributes to injury during ischaemia and is of particular importance in the specialised cells of the proximal tubule in which the cell membrane is augmented by microvilli, which are essential to the normal functioning of these cells. The spectrin–actin cytoskeleton is responsible for the adherence of ion pumps to the cell membrane, and cytoskeletal disruption leads to the redistribution of basolateral Na^+^/K^+^ATPase pumps to the apical membrane within 10 min of cytoskeletal disruption. The resultant bidirectional transport of sodium and water across the apical and basolateral cellular membrane leads to cellular sodium being retransported to the tubular lumen, and thus to an increased fractional excretion of sodium. Effective sodium transport is further disrupted by ineffective transcellular sodium transport, secondary to a deficiency in the supply of ATP. The high concentration of sodium in the filtrate activates glomerular feedback, stimulating the macula densa to induce afferent arteriolar vasoconstriction, with a subsequent reduction in GFR [47]. A diagram summarising the effects of IR injury on the renal tubular epithelium is shown in Fig. 1.

In renal transplants, histological abnormalities associated with delayed graft function due to prolonged ischaemia include increased brush border loss, tubular necrosis, cell shedding, tubular dilatation and interstitial inflammation [51]. The appearance of mild histological abnormalities is often associated with substantial effects on graft function.

Renal dendritic cells and macrophages play an important role in the innate and adaptive immune response in acute IR injury [52] and are also thought to contribute to injury through the production of tumour necrosis factor [53]. Animal models of renal IR injury have shown increased levels of mannin–binding lectin (MBL) early in reperfusion, with subsequent complement deposition [54], although other, more recent animal studies have demonstrated that following reperfusion, the internalisation of MBL by tubular epithelial cells promotes cell death independently of complement activation [55]. Activation of the innate immune system by germline-encoded TLRs in response to renal ischaemia contributes to inflammation and proximal tubular injury, and resolution of this inflammation might be causal in the recovery of injured tubular cells [56, 57]. Upregulation of gene and protein expression of TLR2 and TLR4 in rat kidneys has been demonstrated following IR injury [58] and may be of particular importance to AKI in kidney transplantation.

## Evidence for a clinical benefit of RIPC

Most human studies have used limb ischaemia to activate RIPC due to the inaccessibility of vital organs for IPC. The first such clinical study demonstrated an effect of limb ischaemia to prevent experimental IR injury to the endothelium and was rapidly followed by the first clinical trial of RIPC [59]. In this small study, eight patients undergoing coronary artery bypass grafting (CABG) were randomised to receive either RIPC or control. The results demonstrated an increase in blood lactate dehydrogenase (collected from the coronary perfusion catheter) in the preconditioned group, which the investigators attributed to an ability to maintain anaerobic metabolism in preconditioned cells [59].

In 2007, Hausenloy et al. were the first to demonstrate a reduction in troponin T levels in adults randomised to receive RIPC prior to CABG with cross-clamp fibrillation [60]. In 2009, Venugopal et al. also demonstrated a reduction in troponin T following RIPC in patients undergoing cold blood cardioplegia [61]. However in 2010, Rahman et al. published a larger single-centre double-blind randomised controlled trial in which 162 patients undergoing CABG were randomised to receive either RIPC or placebo. In this study there was no difference in troponin release or in any other clinical outcome between the two groups [49]. Most recently, a larger single-centre study of 329 patients undergoing isolated CABG with cold blood cardioplegia and cardiopulmonary bypass, randomised to RIPC or placebo, demonstrated a reduction in post-operative troponin I in the preconditioned group [62]. The authors also attempted to address the question of whether a reduction in troponin equated to a measurable longer term clinical benefit. They reported a reduction in all-cause mortality in the preconditioned group that was sustained during >4 years of follow-up.

In the clinical setting of primary coronary intervention (PCI) for acute myocardial infarction, Iliodromitis et al. were the first to investigate whether RIPC would attenuate the inflammatory response in elective single vessel PCI with coronary stenting. In their 2006 study, these authors demonstrated an increase in cardiac enzymes and C-reactive protein in the preconditioned group and postulated that RIPC increased the inflammatory response [63]. Subsequently, Hoole et al., in their 2009 study of 242 patients undergoing elective PCI, demonstrated that RIPC prior to PCI attenuated procedure-related troponin release [52]. However, in a separate study, the same group showed that there was no beneficial effect on left ventricular dysfunction during coronary balloon occlusion in single vessel coronary disease [53].

Increased interest in the clinical usefulness of RIPC in the setting of myocardial ischaemia (CABG or PCI) has led to the publication of many other small trials in recent years, all reporting differing outcomes. However, the largest study to date by far—a multicentre double-blind randomised controlled trial, ‘Effect of Remote Ischemic preConditioning on clinical outcomes in patients undergoing Coronary Artery bypass graft surgery’ (ERICCA), is currently underway to investigate whether RIPC improves 1-year cardiovascular outcomes and reduces AKI in the setting of CABG. This trial has recently completed recruitment of 1,610 patients, randomised to either RIPC or sham-RIPC. The primary endpoint is MACCE (=major adverse cardiac and cerebral event(s)—a combined outcome score) at 1 year, but quality of life and echocardiography follow-up are also included [64].

### Evidence for a clinical benefit of RIPC in the kidney

In AKI, there are several interesting clinical questions. Firstly, can RIPC protect the kidneys against ‘bystander’ AKI, i.e. does RIPC prior to myocardial injury reduce collateral damage to the kidney? Secondly, can RIPC protect against planned ischaemic insults such as contrast nephropathy? Thirdly, can RIPC reduce IR injury to the allograft during transplantation? Additionally, can RIPC protect patients with chronic or end-stage kidney disease against IR injury in other organ systems, for example myocardial stunning during haemodialysis?

Animal studies have demonstrated the therapeutic potential of RIPC in protecting from IR injury in the kidney [65, 66], but these benefits have proved difficult to translate into clinical studies in humans. Although several studies have been published in humans, these tend to be small, single-centre studies, and many report differing and short-term endpoints, thus making them difficult to compare or interpret. In addition, the role of co-existent comorbid states and polypharmacy in such patients are confounders, and the degree to which cannot easily be ascertained. A summary of the currently available clinical trial evidence is provided in Table 1, which also presents details on the published trials of RIPC that report renal endpoints.

**Table 1:** Clinical trials of remote ischaemic preconditioning with renal endpoints

Year	*n*	First author	RIPC protocol	Results	Clinical setting
2007	82	Ali [74]	Internal iliac crossclamp, 2 × 10 min	Decreased absolute risk of myocardial injury/ infarction (TnI) and renal injury (creatinine) (*p* = 0.009).	Elective open AAA repair
2009	242	Hoole [75, 76]	Arm, 3 × 5 min	Reduction in TnI at 24 h and MACCE at 6 months. No difference in eGFR or AKI (creatinine >25 % from baseline) at 24-h post-operation. Reduced MACCE maintained at 6 year follow-up (*p* = 0.036).	Elective PCI
2009	40	Walsh [77]	Both legs, 10-min ischaemia	Significantly reduced post-operative urinary RBP (*p* = 0.04). Non-significant reduction in urinary ACR (*p* = 0.06).	Endovascular AAA repair
2010	78	Venugopal [78]	Arm, 3 × 5 min	Post hoc analysis of 2 previous studies. Decreased AKI on AKIN criteria [79] (*p* = 0.005).	CABG
2010	162	Rahman [80]	Arm, 3 × 5 min	No difference in TnT/cardiac performance/inotrope requirement/echo function/arrthymias/renal (peak creatinine/dialysis requiring AKI) or lung outcomes.	CABG (on-pump)
2010	53	Thielmann [81]	Arm, 3 × 5 min	Mean cTnI significantly lower in RIPC group at 6, 12, 24 and 48 h; 44.5 % reduction in cTnI AUC at 72 h. Reduced peak post-operative creatinine (*p* = 0.04).	Elective CABG with crystalloid cardioplegia
2010	40	Walsh [82]	Sequential common iliac clamping	No differences in renal outcomes (urinary RBP and ACR).	Open AAA repair
2011	120	Zimmerman [83]	Leg, 3 × 5 min	Reduced relative risk of AKI (elevation of serum creatinine of ≥0.3 mg/dl or ≥50 within 48 h after surgery), *p* = 0.004.	Cardiac surgery with CPB
2011	113	Pedersen [84]	Leg, 4 × 5 min	No effect on development of AKI (RIFLE criteria), eGFR, urine output or urinary biomarkers (plasma & urinary NGAL, cystatin C).	Paediatric surgery for correction of complex cardiac defects
2011	76	Choi [85]	Leg, 3 × 5 min	Decreased CK-MB at 24 h. No effect on creatinine, cystatin C, NGAL or eGFR.	Complex valvular heart surgery with CPB
2012	70	Hong [86]	Leg, 4 × 5 min carried out pre- and post- anastomoses (RIPC + RPostC)	Reduction in AUC for post-operative TnI of 48.7 % in RIPC/RPostC group. No difference in creatinine or PaO_2_/FiO_2_.	Off pump CABG
2012	54	Kim [87]	Leg, 3 × 10 min pre-,and post-operatively (RIPC + RPostC)	No difference in PaO_2_/FiO_2_, acute lung injury or cytokine release. No difference in creatinine or incidence of AKI (increase in serum creatinine by >50 % or >0.3 mg/dl from baseline within 48 h after surgery).	Complex valvular heart surgery
2012	55	Lee [88]	Leg, 4 × 5 min	No difference in post-operative TnI or peak creatinine.	VSD repair in infants
2012	72	Kottenberg [89]	Arm, 3 × 5 min	Decreased TnI AUC following RIPC administered during isoflurane but not during propofol anaesthaesia. No change in peak postoperative creatinine.	CABG
2012	96	Young [90]	Arm, 3 × 5 min	No difference in post-operative hsTnT or AKI by RIFLE [91] criteria.	‘High risk’ cardiac surgery with CPB
2012	100	Er [68]	Arm, 4 × 5 min	Reduction in risk of contrast nephropathy (increase in serum creatinine ≥25 % or ≥0.5 mg/dL above baseline at 48 h after contrast medium exposure) in patients with pre-existing renal impairment (*p* = 0.002).	Elective coronary angiography
2013	60	Chen [72]	Leg, 3 × 5 min	No difference in urine volumes, urinary biomarkers (MDA, NAG, NGAL, RBP, SOD) or creatinine.	Live donor renal transplantation—randomised to donor/recipient/no RIPC
2013	82	Huang [92]	Leg, 3 × 5 min	1-month GFR significantly reduced in control vs. RIPC group (*p* = 0.034); no difference in GFR at 6 months. Lower 24-h urinary RBP in RIPC group (*p* = 0.001). No difference in serum creatinine or eGFR at 1 or 6 months.	Laparoscopic partial nephrectomy for renal tumour
2013	60	Igarashi [93]	Arm, 4 × 5 min	% change in urinary L-FABP level at 24 h was significantly smaller in the RIPC group (*p* = 0.003).	Elective coronary angiography in patients with MDRD eGFR 30–60 ml/min/1.73 m^2^

AAA, Abdominal aortic aneurysm; ACR, albumin:creatinine ratio; AKI, acute kidney injury; AKIN, Acute Kidney Injury Network; AUC, area under the curve; CABG, coronary artery bypass grafting; CK-MB, creatine kinase muscle brain type; CPB, cardiopulmonary bypass; (e) GFR, (estimated) glomerular filtration rate; L-FABP, liver-type fatty acid binding protein, MACCE, major adverse cardiac and cerebral event; MDA, malondialdehyde; MDRD, modification of diet in renal disease; NAG, *N*-acetyl-D-glucosaminidase; NGAL, neutrophil gelatinase associated lipocalin; PaO_2_/FiO_2_, partial pressure of oxygen in arterial blood /fraction of inspired oxygen; PCI, percutaneous coronary intervention; RBP, retinol binding protein; RIFLE, Risk Injury Failure Loss End-stage kidney disease; RIPC, remote ischaemic preconditioning; RPostC, remote ischaemic postconditioning; SOD, superoxide dismutase; TnI, troponin I; (hs) TnT, (high sensitivity) troponin

### Clinical trials of RIPC to protect against bystander AKI

A recent meta-analysis of studies in cardiac/abdominal aortic aneurysm surgery suggests that there is a benefit of RIPC in reducing renal injury post-surgery [67]. However in only five trials were the absolute creatinine values documented and included in the analyses; however, differing measures were reported and so the results were adjusted and reported as standardised mean values. Additionally, these trials were not powered towards renal endpoints, and the total number of patients included was 377, which is still most likely underpowered to detect a significant renal effect. The doubt over whether RIPC can protect against bystander renal injury during surgery should be answered in due course by currently ongoing large clinical trials, such as ERICCA.

### RIPC in protection against contrast nephropathy

One other potential application that has been investigated in a clinical trial is the use of RIPC to protect against contrast-induced AKI. Patients with pre-existing renal dysfunction (serum creatinine >1.4 mg/dl or estimated (e) GFR of <60 ml/min/1.73 m^2^) were randomised to receive RIPC (4 × 5-min arm cuff inflations) or sham prior to elective coronary angioplasty. The authors reported a reduction in the rate of contrast-induced AKI, from 40 % in the control group to 12 % in the RIPC group (*n* = 100, *p* = 0.002) [68]. Not only are these results of potential relevance in the setting of CKD patients undergoing routine investigation or elective angiography, especially in avoiding the precipitation of dialysis dependence in patients with CKD stage 5 estimated GFR [(eGFR) <15 ml/min], but they may also help to relieve anxiety surrounding the use of PCI in patients presenting acutely. Studies have demonstrated increased morbidity and mortality in patients with CKD who present with acute coronary syndromes, some of which may be attributable to management possibly being compromised by a reluctance to use therapies involving contrast, which may subsequently precipitate dialysis [69].

A further single-centre randomised controlled trial, ‘Effect of Remote Ischaemic Conditioning on Contrast-Induced Nephropathy in patients undergoing elective coronary angiography (ERICCIN)’, is currently underway. The aim of this study is to recruit 362 patients at risk of contrast nephropathy (pre-existing eGFR <60 ml/min/1.73 m^3^), randomised to either 4 cycles of 5 min of arm cuff inflation to 200 mmHg or sham (10 mmHg), administered 2 h prior to contrast administration for cardiac catheterisation. The primary endpoint is a rise in creatinine of >25 % of eGFR, or a rise in creatinine of >44 μmol/l at 48 h, with secondary endpoints of eGFR over 3 months and the biomarkers neutrophil gelatinase-associated lipocalin (NGAL) and urinary albumin at 6 h, 48 h and 3 months post-contrast administration [70].

### RIPC in kidney transplantation

The use of direct IPC in transplantation (preconditioning of the donor organ at retrieval by repeated clamping/unclamping of the arterial supply) has been investigated in clinical trials in liver transplantation [71]. However, no similar studies have as yet been published in kidney transplantation.

A pilot clinical trial carried out by our group in the setting of paediatric living-donor renal transplantation demonstrated the protective effects of late (‘second window’) RIPC. A blood pressure cuff was used to cause 5-min periods of limb ischaemia (3 cycles, applied to the donor and recipient) 24 h in advance of surgery. A prospective cohort of patients (*n* = 20) were randomised in a blinded fashion to sham RIPC or RIPC (*n* = 10 in each group). The groups did not differ in terms of donor/recipient age, sex, weight, height, baseline creatinine or cold ischaemic time.

**Fig. 2:**
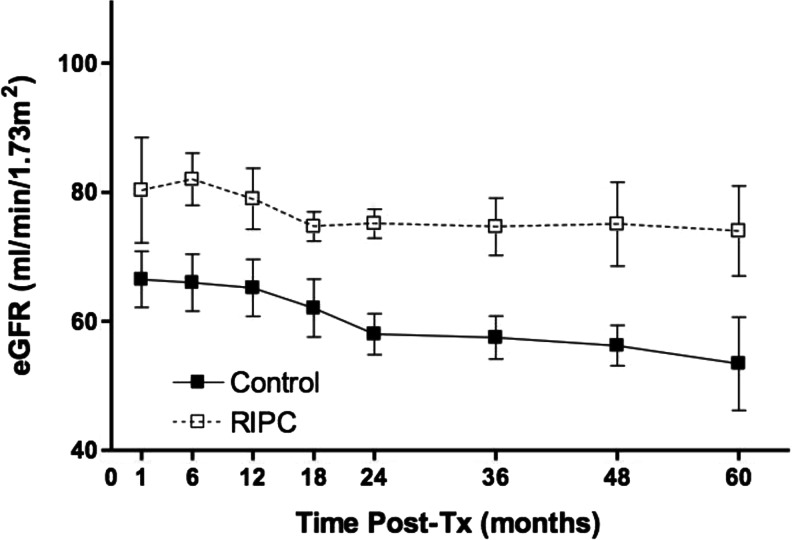
Effect of remote ischaemic preconditioning (*RIPC*) on long-term graft function following transplantation (*Tx*). *eGFR* Estimated glomerular filtration rate

Post-operative excretion of retinol binding protein (RBP; area under the curve for RBP 72 h post-transplantation) in RIPC patients was significantly reduced compared to the controls [1.2 × 10^5^ vs. 1.5 × 10^5^, respectively; *p* = 0.02]. The time for plasma creatinine to halve was shorter in the RIPC group than in the controls (5.5 ± 2.3 vs. 9.4 ± 3.5, respectively; *p* = 0.007). RIPC resulted in significant improvement of long-term renal function. The mean area under the curve (AUC eGFR) for the intermediate follow-up period (1–24 months post-transplantation) was 366 and 303 in the control (*n* = 10) and RIPC (*n* = 9) groups, respectively (*p* = 0.009). The AUC eGFR for the late follow-up period (up to 60 months post-transplantation) was also significantly higher in the RIPC group (1,190; *n* = 7] than in the control group (1,103; *n* = 7; *p* = 0.04) (Fig. 2).

A second randomised controlled study of RIPC in renal transplantation was published (as a letter to the editor) earlier this year. In this small study, live donor kidney transplant recipients and their donors were randomised in pairs to receive either donor RIPC, recipient RIPC or none. The RIPC stimulus was 3 × 5-min leg cuff inflations to 300 mmHg, separated by 5 min of reperfusion. The timing of the RIPC stimulus prior to surgery was not specified. In this small study, the authors did not observe any differences between the three groups in terms of urine volume, plasma creatinine, AKI biomarkers, length of hospital stay or cost [72].

Of note, a study of direct ischaemic preconditioning in the setting of deceased-donor transplantation has reported a significant improvement in creatinine, eGFR and urine NGAL in patients who received the intervention. In this study, perconditioning (i.e. ischaemic conditioning performed during the period of ischaemic injury) was delivered by 3 × 5-min cycles of external iliac artery clamping, carried out while the venous and arterial anastomoses were formed [73].

A much larger study of RIPC in live donor renal transplantation, the REnal Protection Against Ischaemia Reperfusion in transplantation (REPAIR), has finished recruiting and completed 1 year of follow-up for the primary endpoint (iohexol GFR at 12 months post-transplantation). This study is unique in that it examines whether there is an additive effect of early and late preconditioning—participants are randomised in donor/recipient pairs to receive early RIPC, late RIPC, both or none. A study of RIPC immediately prior to surgery in cadaveric renal transplantation (recipient RIPC) is also underway in Scandinavia, and the Remote Ischemic Preconditioning in Abdominal Organ Transplantation (RIPCOT) study is recruiting 580 deceased organ donors and recipients of kidneys, livers and pancreata.

It is hoped that the results of these trials will determine whether RIPC should be accepted as a standard part of care in renal transplantation. Further questions, such as whether RIPC should be applied to the donor, recipient or both, will need to be directly addressed in subsequent studies.

### Other potential applications of RIPC

Other potential benefits of RIC during native AKI, such as to improve renal function, reduce mortality or protect against long-term CKD, have not as yet been investigated. It is challenging to conduct studies in this setting in that the onset, degree and nature of the IR injury cannot be predicted and, therefore, the comparison of cohorts in clinical trials would prove difficult.

A clinical trial is currently underway investigating the effects of RIPC to protect against myocardial stunning in haemodialysis patients. The primary endpoint is regional wall abnormalities on two-dimensional echocardiogram within 4 h of the intervention, with secondary endpoints including: change in haemodynamic variables, frequency of intradialytic hypotension, longer term echocardiographic changes and the biomarkers troponin-T, plasma interleukin-6 and N-type pro-brain natriuretic peptide.

## Summary

Remote ischaemic preconditioning is a safe, inexpensive and well-tolerated intervention that might have significant clinical benefits in reducing tissue and organ damage following IR injury. Several large randomised controlled clinical trials are currently underway which should resolve current conflicts regarding the potential utility of this technique in clinical practice.
